# Machine learning powered tools for automated analysis of muscle sympathetic nerve activity recordings

**DOI:** 10.14814/phy2.14996

**Published:** 2021-08-24

**Authors:** Janis M. Nolde, Leslie Marisol Lugo‐Gavidia, Revathy Carnagarin, Omar Azzam, Márcio Galindo Kiuchi, Ajmal Mian, Markus P. Schlaich

**Affiliations:** ^1^ Dobney Hypertension Centre School of Medicine ‐ Royal Perth Hospital Research Foundation Faculty of Medicine Dentistry & Health Sciences The University of Western Australia Perth Australia; ^2^ School of Computer Science and Software Engineering The University of Western Australia Perth Australia; ^3^ Departments of Cardiology and Nephrology Royal Perth Hospital Perth Australia; ^4^ Neurovascular Hypertension & Kidney Disease Laboratory Baker Heart and Diabetes Institute Melbourne Australia

**Keywords:** algorithms, artificial intelligence, hypertension, machine learning, microneurography, muscle sympathetic nerve activity, sympathetic nervous system

## Abstract

Automated analysis and quantification of physiological signals in clinical practice and medical research can reduce manual labor, increase efficiency, and provide more objective, reproducible results. To build a novel platform for the analysis of muscle sympathetic nerve activity (MSNA), we employed state‐of‐the‐art data processing and machine learning applications. Data processing methods for integrated MSNA recordings were developed to evaluate signals regarding the overall quality of the signal, the validity of individual signal peaks regarding the potential to be MSNA bursts and the timing of their occurrence. An overall probability score was derived from this flexible platform to evaluate each individual signal peak automatically. Overall, three deep neural networks were designed and trained to validate individual signal peaks randomly sampled from recordings representing only electrical noise and valid microneurography recordings. A novel data processing method for the whole signal was developed to differentiate between periods of valid MSNA signal recordings and periods in which the signal was not available or lost due to involuntary movement of the recording electrode. A probabilistic model for timing of the signal bursts was implemented as part of the system. Machine Learning algorithms and data processing tools were implemented to replicate the complex decision‐making process of manual MSNA analysis. Validation of manual MSNA analysis including intra‐ and inter‐rater validity and a comparison with automated MSNA tools is required. The developed toolbox for automated MSNA analysis can be extended in a flexible way to include algorithms based on other datasets.

## Graphical abstract

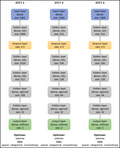

## 1 INTRODUCTION

More than half a century ago the introduction of microneurography led to the realization that the activity of distinct parts of the nervous system can be directly recorded and quantified (Carter, 2019). This technique granted direct access to the activity of the sympathetic nervous system, an important controller of cardiovascular regulation. Microneurography enables the direct measurement of postganglionic efferent sympathetic nerve activity directed to the skeletal muscle vasculature (MSNA). The sympathetic nervous system – responsible for stress responses and maintenance of a wide variety of autonomic physiological functions – from blood pressure and heart rate to visual acuity to digestion – has also been found to be of significant pathophysiological relevance in many medical conditions (McCorry, 2007). including hypertension (Grassi, 1998; Hering et al., 2014; Schlaich et al., 2004), congestive heart failure (Azevedo et al., 2020; Schlaich et al., 2005), left ventricular hypertrophy (Schlaich et al., 2003), ischemic heart disease (Badrov et al., 2016; Malliani & Montano, 2004), Takutsubo cardiomyopathy (Vaccaro et al., 2014), atrial fibrillation and sudden cardiac death (Kiuchi et al., 2019), sleep apnea (Floras, 2009), chronic kidney disease (Kaur et al., 2017; Schlaich et al., 2009), and obesity (Lambert et al., 2014). Importantly, increased SNS activity has been shown to predict CV outcomes independent of other risk factors and its inhibition is considered a key therapeutic approach in many of these conditions.

MSNA is recorded by insertion of a fine‐insulated electrode into a peripheral nerve, most commonly the peroneal nerve, which is quite superficial and located just below the head of the fibula which serves as an anatomic landmark. If the tip of the electrode is appropriately placed within the efferent sympathetic nerve fiber, the degree of central sympathetic outflow directed to the post‐ganglionic skeletal muscle vasculature can be recorded as an integration of fiber activity (bursts) or even single nerve recordings (spikes). These signals commonly undergo pre‐processing before analysis, including amplification, filtering, and integration (White et al., 2015). This results in signals featuring characteristically shaped bursts of sympathetic activity, which can be identified and quantified in terms of their frequency (burst frequency expressed as bursts/min) and burst incidence (expressed as bursts per 100 heartbeats) and total MSNA. (White et al., 2015). These parameters have proven to be of high scientific value in scientific physiological and pathophysiological considerations as indicated by the wide range of research efforts mentioned above.

However, the process of MSNA signal analysis has some substantial caveats that need to be taken into consideration. The processed signals tend to be noisy even after integration, with considerable inter‐measurement differences of signal quality. Some appear very clean with stable baseline tracings and distinct typically shaped signal peaks representing sympathetic bursts. Others feature very unclear baselines and heterogeneously shaped electrical activity in which typical signals are still identifiable, but a clear differentiation between valid representations of MSNA and noise remains highly subjective (White et al., 2015). An excellent technical review by White et al. revisits possibilities for standardization of this process such as establishing a height offset of valid signals from the baseline by a relation of 3:1–without negating potential disadvantages of these approaches. Of importance, the analysis of these signals is subject to a potentially strong individual bias of the interpretation of the signals especially when the signal is very noisy. White et al. point out that there are no data investigating the potential impact of differing methods and approaches for the identification of MSNA bursts.

Additionally, scientists can make use of multiple markers while analyzing MSNA recordings to identify valid signal bursts and their timing. The initiation of sympathetic bursts occurs in the brainstem usually when the blood pressure is at its lowest point–during the diastole of the cardiac cycle. Taking these physiological insights consistently into account may help to improve the appropriate and accurate analysis of MSNA and reduce the potential of error.

Furthermore, accessing the nerve and positioning the recording electrode within the nerve fiber often takes a considerable amount of time, whereas the smallest of movements by the subject can lead to the loss of a valid MSNA signal. Manually marking digital recordings as well as retrospective review of the signal quality are needed to identify valid MSNA recording periods that can be used for analysis. Since MSNA recordings can last up to hours, their interpretation and analysis takes time and concentration. Some authors use peak detection software to simplify and speed up this process. These types of software however, often cannot correctly differentiate noisy from valid signals nor identify high quality recording windows to focus the subsequent analysis on such windows. Therefore, the development of tools to further standardize, digitalize, and automate this process is a logical step to move the technique of MSNA analysis forward.

To achieve this goal, appropriate analysis tools would need to take our domain knowledge about valid MSNA signals into account, in the first instance this might include:

1. Shape and form of signal peaks and immediate surrounding that are regularly regarded as valid MSNA bursts
2. Timing of the signals in regards to other physiological markers of the cardiac cycle
3. Overall signal quality and detection of relevant and irrelevant time periods (i.e., stable high quality signal vs. signal during electrode positioning/adjustment associated with noise and artifacts)

Technological advances mostly in hardware and software have prompted the usage and further development of fast automated data processing tools that make these tasks achievable (Donoho, 2017). The possibility for machine learning algorithms to differentiate patterns, shapes and forms makes it one of the most promising methods to facilitate interpretation of complex physiological signals. We hypothesize that neural networks which have shown to be particularly powerful at pattern recognition can be trained with labeled datasets to learn to differentiate valid MSNA signals (bursts) from signal peaks that are more likely to represent noise or other activity (Bermejo et al., 2019). Since this is the first study to our knowledge exploring the potential of AI applications for MSNA recordings, we furthermore wanted to explore different approaches to the problem of identifying valid MSNA signal bursts. This included different machine learning training approaches, different labeling strategies of the data and evaluation of supplementary features such as burst timing and overall signal‐quality.

MSNA burst timing is associated with some uncertainty, however, typically occurs at a specific time of the cardiac cycle which is often taken into account when interpreting microneurography results. Probabilistic models marking areas of higher likelihood for valid MSNA burst based on the cardiac cycle (measured by ECG and continuous blood pressure measurement) might prove to be helpful in modeling the overall likelihood of a signal peak to represent a valid MSNA burst. We set out to explore the quantitative temporal relationship between measures of the cardiac cycle and use the continuous data indicating the stages of the cardiac cycle for a computational model that assigns varying likelihood densities to certain time points during the microneurography recording based on values found for this relationship in the literature.

Automated quality parameters for MSNA signals have not been described in the existing scientific literature to the best of our knowledge. The goal was to develop a quantitative marker that would differentiate, as clearly as possible, between a generally valid signal and a non‐valid signal. That means in a best‐case scenario that this marker would reliably identify a change in signal quality after appropriate positioning of the electrode in the nerve fiber. This would distinguish valid recordings from time points at which the electrode is manipulated to obtain an adequate signal.

The overarching goal was to implement the necessary algorithms and models in one program that took raw files of the signal data as input and produced markers for all detected signal peaks that represent the aforementioned quality parameters. As a consequence, the data can be easily analyzed based on the likelihood assigned to the individual signal peaks and recording periods to represent valid MSNA signals.

## 2 MATERIALS AND METHODS

### 2.1 MSNA, ECG, and Blood pressure recordings

The data used for the analysis and development of algorithms described here was based on data recorded in either young (18–30 years old, *n* = 24) or older age healthy male individuals (60–75 years old, *n* = 10). When only a part of the data was used for certain aspects of analysis or algorithm development, this is indicated in the individual sections. In such cases, the selected recordings were chosen randomly.

In all subjects multiunit postganglionic MSNA was recorded with tungsten microelectrodes (FHC, Bowdoinham, ME) that were inserted directly into the right peroneal nerve below the fibular head. Blood pressure was continuously measured with the Finometer system (Finapress Medical System BV, Amsterdam, The Netherlands). An ECG was recorded with a 5 lead system with all other described parameters at a sampling rate of 1000 data points per second (PowerLab recording system, model ML 785/8SP, ADI Instruments, Bella Vista, NSW, Australia). Before recording, the data was integrated by an analog transistor with data processing capacity in 100 ms intervals. Data were recorded after finding a valid signal for a prolonged period of usually 90 min. Data were exported as text files for further processing.

The study was approved by the Ethics Committee of the University of Western Australia and abides by the declaration of Helsinki.

### 2.2 Neural networks for MSNA signal shape detection

For training and testing of neural networks, three datasets (Dataset 1, Dataset 2, and Dataset 3) consisting of images of MSNA signals were labeled in terms of their likelihood of one central, marked, signal representing a valid MSNA signal. The datasets consisted of 1000, 1100, and 1900 signals. In each dataset, 100 images represented data from one individual participant in whom microneurography was performed. In other words, for the first dataset with 1000 images, 100 images per participant of a total of 10 participants were used. Images were created for each peak detected with an ordinary peak detection algorithm calibrated such that it included a broad peak detection (width: 100 ms, distance; 250 ms, height minimum: 0.05, maximum: 0.8, prominence = 0.05 V). Of these images, 100 were randomly selected per participant and included in the Dataset 1. Datasets 2 and 3 were arranged in a similar manner, with the data of 11 and 19 participants randomly selected from the entire dataset and both age groups, respectively. Furthermore, datasets 2 and 3 were prescreened by the neural network created from dataset 1 for likelihood to represent valid MSNA signals. The random selection of data from patients for datasets 2 and 3 was calibrated based on this to include 60% of peaks that were found to likely be valid MSNA signals by the first neural network and the remaining 40% likely to be non‐MSNA signals. All signal peaks were sampled randomly from the whole recording, including substantial periods of preparation and needle manipulation to find the signal. The sampled signals were therefore highly heterogeneous and noisy.

Labeling was performed for each dataset with slightly different premises to diversify the information available to the labeling procedure in each dataset. Dataset 1 was labeled with showing 20 s excerpts of MSNA, blood pressure and ECG recording and marking the relevant peak in the signal in terms of the rating. The marked peak was then rated on a binary scale as either a valid or invalid signal in all datasets. Dataset 2 was labeled showing the rater only a 1‐s window of the signal peak of the MSNA recording. Dataset 3 was labeled based on a 1‐s and a 4‐s window of the MSNA recording available for each signal peak. For all visualizations, the peak that was to be rated was clearly marked and positioned in the exact center of the image. Ratings were carried out by a medical doctor with regular training in interpretation of microneurography signals. Table 1 summarizes the datasets used to train the neural networks. The manual labeling of the data followed as many standard criteria for microneurography analysis as feasible, a process that was partially limited by the fact that only individual signal bursts were rated and only parts of the data were deliberately selected for labeling of the three different datasets. Where applicable, a 3:1 signal to noise ratio was employed and timing in relation to the cardiac cycle was utilized when this was available for rating as described previously (White et al., 2015).

**TABLE 1:** Dataset characteristics

	DTCT 1	DTCT 2	DTCT 3
Signal samples	1000	1100	1900
Participants	10	11	19
Signal choice	Random	Based on DTCT 1 predictions to include 60% valid signals	Based on DTCT 1 predictions to include 60% valid signals
Signal information presented to rater	20‐s windows of microneurography recording, blood pressure, and ECG	1‐s window of microneurography	1‐s and 4‐s window of microneurography recording
Marking of signal to be rated	Central position in window and visual marking	Central position in window and visual marking	Central position in window and visual marking
Rated signal	One per excerpt	One per excerpt	One per excerpt
Signal used for neural network training	4 s around maximum of rated signal	4 s around maximum of rated signal	4 s around maximum of rated signal

**FIGURE 1:**
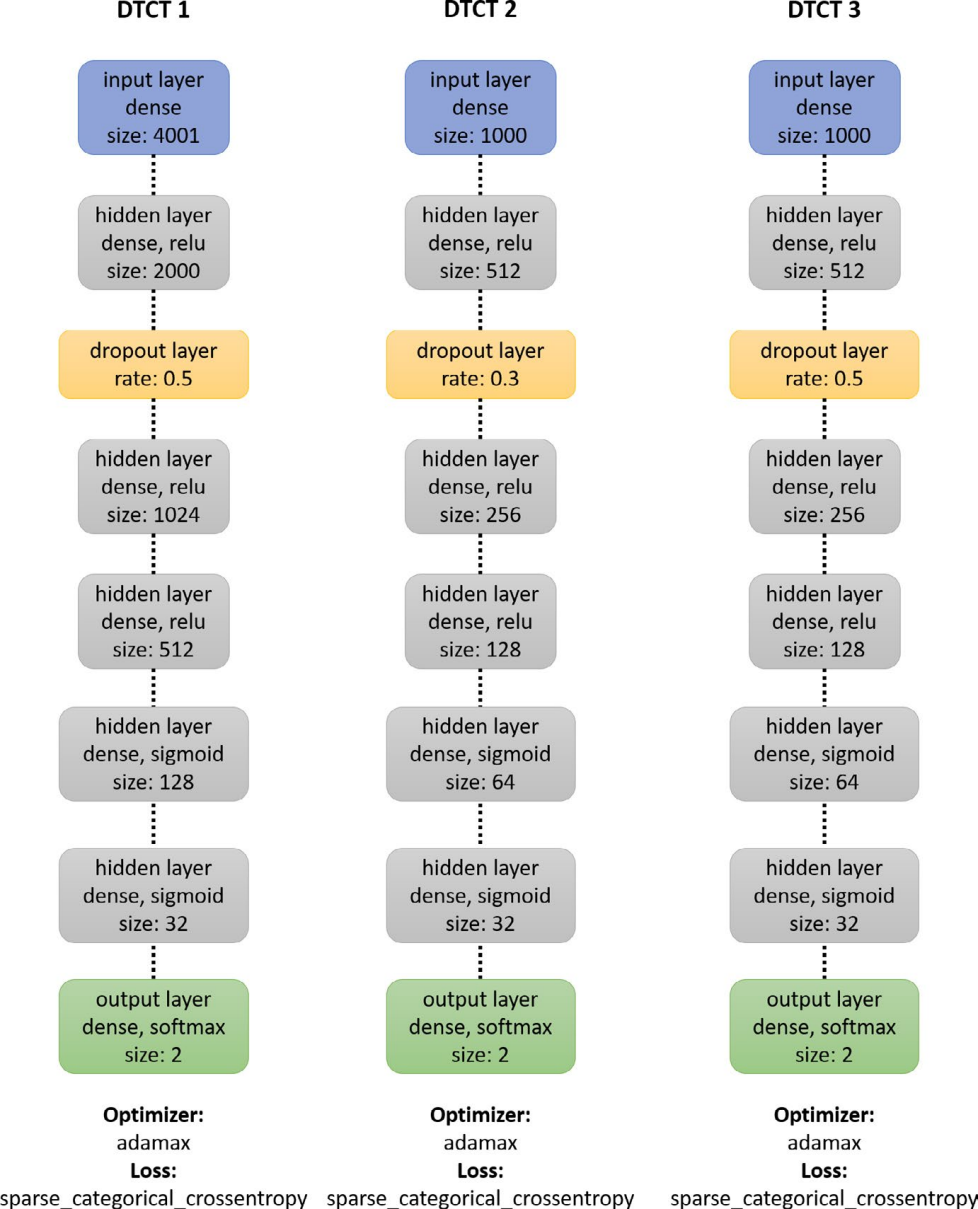
Architecture of neural networks. The first line in each box refers to the type of layer, the second specifies further properties and the activation settings of the individual layer (Tensorflow activations relu, sigmoid, and softmax were employed), and the last line specifies the dimensions of the layer if applicable

Neural networks were trained using the one‐dimensional vectors of the MSNA signal windows as input data and the binary rating as output variables. For the first network, 4 s excerpts were chosen around the central point of the signal (center minus and plus 2000 ms at a sample rate of 1 per ms), resulting in an input vector of 4001 data points. For neural Network 2 and 3, only 1s excerpts were used, resulting in one dimensional input vectors with a length of 1000 ms. See Figure 1 for detailed visualizations of the individual algorithms. Algorithms were built on Ubuntu 18.04 server environment in python with Keras using the Tensorflow deep learning library.

The datasets were separated into training (80%) and testing (20%) splits. In the training partition of Dataset 2, positively rated signals were triplicated to account for the low numbers of positive samples (which led to an overall training dataset size of 1492 and testing dataset size of (220). All algorithms were trained from initiation 100 times after random reshuffling of the data into train and testing sets. The models were reset after each iteration and completely retrained with the newly split and reshuffled data. For the first algorithm (DTCT 1) a batch size of 32 and 6 epochs were used, for the second (DTCT 2) the batch size was set to 32 with 20 epochs and the third algorithm (DTCT 3) was trained in batches of 16 over 35 epochs. All algorithms were trained using Tensorflow “adamax” optimizer and “sparse categorical crossentropy” as the loss function. A dropout layer was added the neural networks, which had consisted of five hidden layers each. A graphical illustration of the detailed neural network architecture is provided in Figure 1. No substantial model finding or tuning process was employed. The described models were essentially identical with our initial attempts. The amount of epochs were varied between the different approaches to explore whether training performance would further increase, but models appeared to reach optimal performance within a limited amount of training epochs. Performance values such as accuracy and area under the receiver operating curve (ROC AUC) were recorded for each iteration and average learning curves and loss curves created based on the test dataset unseen by the algorithm until then. The last version of the algorithm that was trained was used for all further applications.

### 2.3 Signal timing–association of MSNA‐Signals, Blood pressure and ECG

**FIGURE 2:**
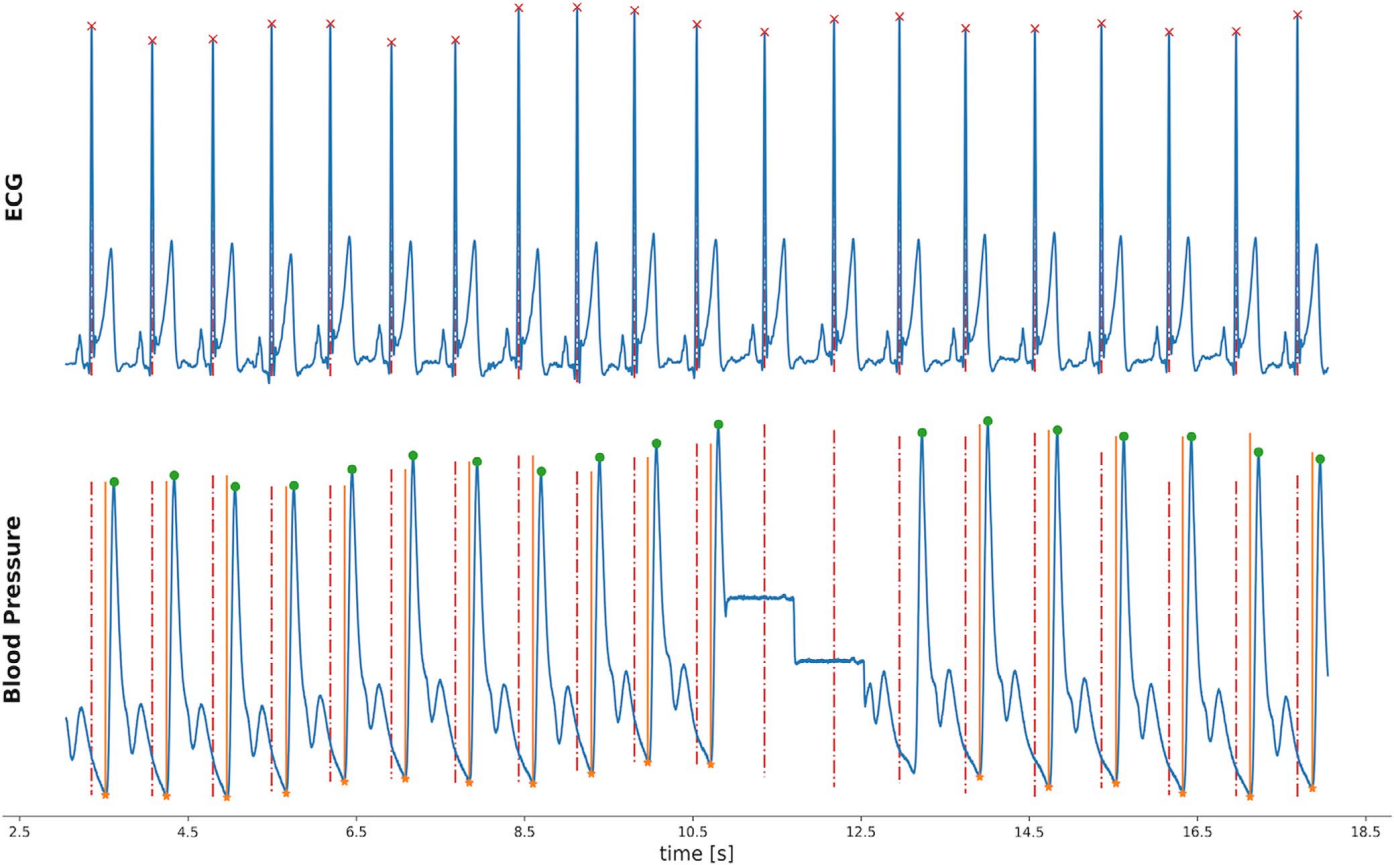
Visualization of the quanitfication process between ECG and BP offset. ECG R‐waves are marked with red crosses and interrupted lines in the upper panel and interrupted lines in the lower panel. The lowest point of the BP cycle is marked in the lower panel with an orange dot and line, the highest with a green dot. Temporal difference between red and orange line was assessed in over 100,000 instances

For development of a probability estimate for the timing of the MSNA signal, associations with continuous blood pressure measurements and ECG were examined. Since continuous blood‐pressure recordings tend to have artifacts and interruptions, we used the regular relation between blood pressure and ECG signal–given that the patients are healthy and have no arrhythmias–for this estimate. We sampled 98,208 heartbeats as ECG and blood pressure recordings from continuous recordings over multiple hours taken from 34 patients. The data were selected by marking the local blood pressure minima (diastolic BP–orange dots and lines in Figure 1) and matching them with the closest ECG local maximum (QRS signal in ECG–red crosses and interrupted lines in Figure 2). To exclude artifacts in the continuous blood pressure measurements, local BP minimal were excluded if they were not followed by a local BP maximum within 150 ms (distance between orange dot/line and green dot in Figure 2). Data that were unlikely to represent valid relations between ECG and blood pressure data of the same heart beat (due to artifacts or gaps in the recording) were excluded. Such data were identified by calculating a *z*‐score for each data point based on the given distribution (see Figure 9b for an excerpt of this first distribution with a long tail of few, very large differences–overall 109,719 data points were included in this distribution). Data points with a higher z‐score than 0.12 were excluded. The data were furthermore clipped at −250 ms, discarding the values lower than that to focus on the dominant distribution (long negative tail). This led to focusing the centered, dominant distribution see in Figure 9a of 98,208 differences between local ECG maxima and blood pressure minima.

The resulting median of this distribution was used as the offset of the time point for the lowest blood pressure (diastole) from the ECG signal (ECG signal local maximum +median of ECG and blood pressure difference). This relation made it possible to connect the physiologically interlinked lowest point of blood pressure and likelihood of occurrence for MSNA signal to the timing of the ECG signal and use it even if the blood pressure signal is not available due to artifacts. The delay of MSNA signals and the cardiac cycle (ECG in this case) has been measured to be within the range of 1160 and 1490 ms with a strong dependence of body length (Sundlöf & Wallin, 1978). Later studies confirmed this temporal offset also for blood pressure measurements, for which they found an average offset of 1240 ms (Hissen et al., 2015). Other studies applied intervals for the offset of usually 1200–1400 ms (Kienbaum et al., 2001). For our application, a body height adjusted approach based on the method reported by Sundlöf et al. appeared to be the most precise approach. A regression model based on the line of best fit shown by Sundlöf et al. in 1978 was used to calculate the most likely offset to the ECG R‐peak, a default value of 175 cm was used in case no height data were available. This time was subtracted from the time point of the individual MSNA signal‐peaks and a normally distributed probability distribution with a standard deviation of 200 ms centered around the corresponding time point that resulted from this subtraction. The next ECG R‐wave to the center of this distribution was assessed with its position in this probability distribution and the corresponding MSNA signal labeled with the probability density at the point of the ECG signal within this distribution. The resulting probability densities for each MSNA signal‐peak were normalized to a scale of 0 to 1 for the final output.

### 2.4 Markers for signal quality quantification

**FIGURE 3:**
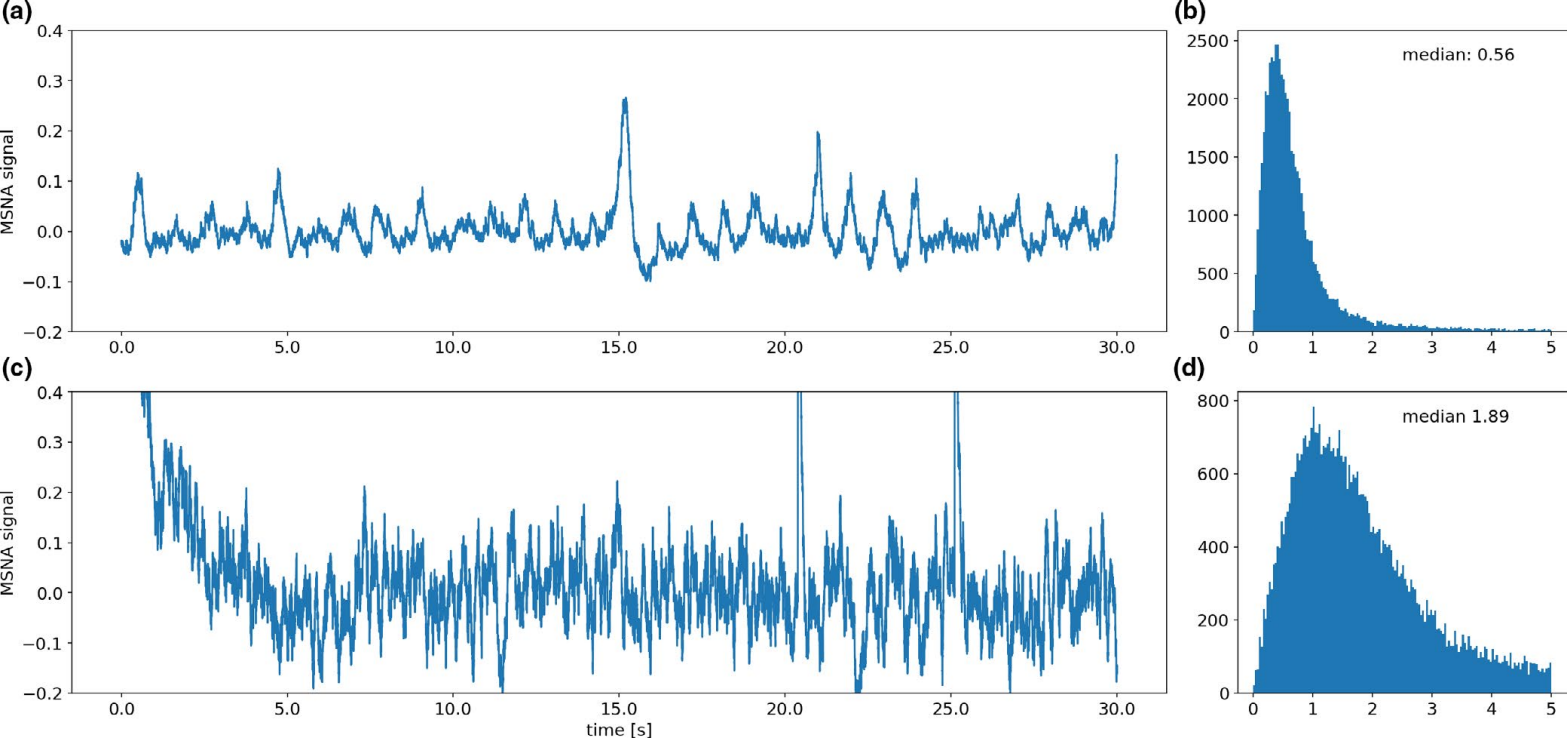
Two excerpts of the microneurography recording in one participant. The upper panels show the recording after finding a valid MSNA signal (a, only the first 30 s of the 60 s excerpt on which Fourier transformation was carried out is displayed) and the histogram of the one dimensional discrete Fourier transformation of a 60 s except with the x‐axis just showing the absolute frequency of individual values (b). Panels (c) and (d) show the same analysis for an excerpt at the very beginning of the recording when no valid signal had been acquired yet

For quantification of different levels of signal quality, simple one‐dimensional discrete Fourier transformation of the raw data was performed. This rendered distinct distributions in the frequency of values in the absolute (negative values were changed into positive ones) transformed data depending on the overall signal quality. In general, noisy signals in which valid MSNA signals were absent led to broader distributions with a stable median of roughly ≥1.5 (see Figure 3c and d), while cleaner signals with valid MSNA signal candidates rendered narrow distributions with typically distinctly lower medians (see Figure 3a and b).

This method was applied to a dataset of 1000 excerpts of 60 s, of which the central 30 s had been rated to be either noisy signal without valid MSNA signal candidates (Figure 3c) or to signals with little noise and valid MSNA signal candidates (Figure 3a). In between group differences were visualized and tested for statistical significance. For visualization purposes, boxplots were used with a centered horizontal line representing the median. Borders of the boxes represent the interquartile ranges (25% and 75%), notches represent bootstrapped 95% confidence intervals (10,000 resamples), whiskers and circles represent outlier data Figure 11. Only 990 of 1000 excerpts could be analyzed as 10 samples were too close to the end or beginning of the recording to extend them from the displayed 30 s excerpt to the analyzed 60 s. The median of the samples representing valid MSNAs signal excerpts was used as the center of a normal‐likelihood distribution with a standard deviation of 0.3.

### 2.5 Implementation

Raw data from MSNA recordings with a sample rate of 1000 data points per second was saved as text files and imported into a python3 environment. A simple peak detection algorithm (Taskesen, 2020) was used to find all local prominences of more than 0.05 Volts prominence and 100 ms width (furthermore the height was set to a minimum of 0.05 and maximum of 0.8 Volts, the distance function of the algorithm was set to 250 ms). For each detected peak distance to the closest ECG signal was calculated and used to assign a likelihood according to the likelihood distribution (normally distributed) created for the ECG–BP relation centered around 180 ms after the ECG peak (R‐wave) with a standard deviation of 200 ms. This likelihood was scaled from 0 to 1. For each peak an excerpt corresponding to the input layer size of the trained network was selected as input for each of the three neural network algorithms to assess the likelihood of a valid MSNA peak. This likelihood was given as a likelihood for a negative outcome and a positive outcome each on the scale from 0 to 1 and one categorical value that was selected based on which likelihood was higher as either negative or positive for each algorithm. One‐dimensional discrete Fourier transformation was then performed on a 60 s window around each detected peak of the MSNA signal and the median of this distribution was calculated. This median was associated with the individual signal as its main signal quality marker. This quality index value of the individual signal peaks and their 60 s surrounding was assessed with the likelihood distribution created for this quality index and the corresponding probability density was assigned to the particular signal‐peak. All assigned probability densities for each signal around a detected peak were scaled from 0 to 1.

Each of these variables for individual signal validity (neural networks), timing (MSNA‐ECG‐BP association), and signal Quality (likelihood of one‐dimensional discrete Fourier transformation) could now be associated with an individual detected peak, and all variables were scaled between 0 and 1, some of them continuously and some as a (binary) categorical variables. All variables were then plotted as a line plot, the continuous likelihoods from the neural networks for a positive outcome were plotted left and right of their categorical outcome, 0 to 1 scaled quality and timing likelihood indexes were plotted thereafter. The line plots were transformed into an axially orientated, round plots, and arranged in a way that each dimension of assessment was represented in a different area of the circle so that the shape of the circle could immediately confer after some training a representation of the quantified variables of each individual signal peak (see Figure 12, Figure 13, and Figure 14. For the default form of this circular representation, the quality index was set to occupy the largest proportion of the circle and timing and the individual neural network outputs the other half. Integration of this curve and scaling of the area under the curve (AUC) led to an overall composite of all quantified markers from 0 to 1 representing an overall likelihood for the validity of each individual peak in the MSNA signal. The scaling was applied to ensure that the entire surface of the plot would exactly add up to be equivalent to the numerical value of one (so that the maximal AUC would be one). This was achieved by dividing the actual AUC below this line by the overall surface of the plot.

## 3 RESULTS

### 3.1 Neural networks for MSNA signal shape detection

#### 3.1.1 First neural Network DTCT 1

**FIGURE 4:**
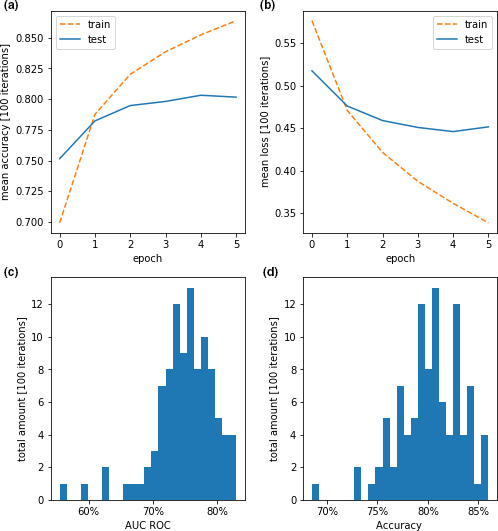
Performance parameters for the training for DTCT 1. Panels (a) and (b) show the average accuracy and loss of 100 individual, completely separated training repetitions of the algorithm for training and testing dataset. Panels (c) and (d) show histograms of the performance parameters AUC ROC and accuracy for each individual time the algorithm was trained

In 100 independent training sessions, the first neural network achieved on average 80.54% accuracy on the testing dataset and a mean ROC AUC of 75.0%. Visualizations of the training and performance parameters can be found in Figure 4, with average learning and loss curve over the epochs and histograms for accuracy and AUC ROC over 100 independent training sessions with each reshuffled data.

#### 3.1.2 Second neural network DTCT 2

**FIGURE 5:**
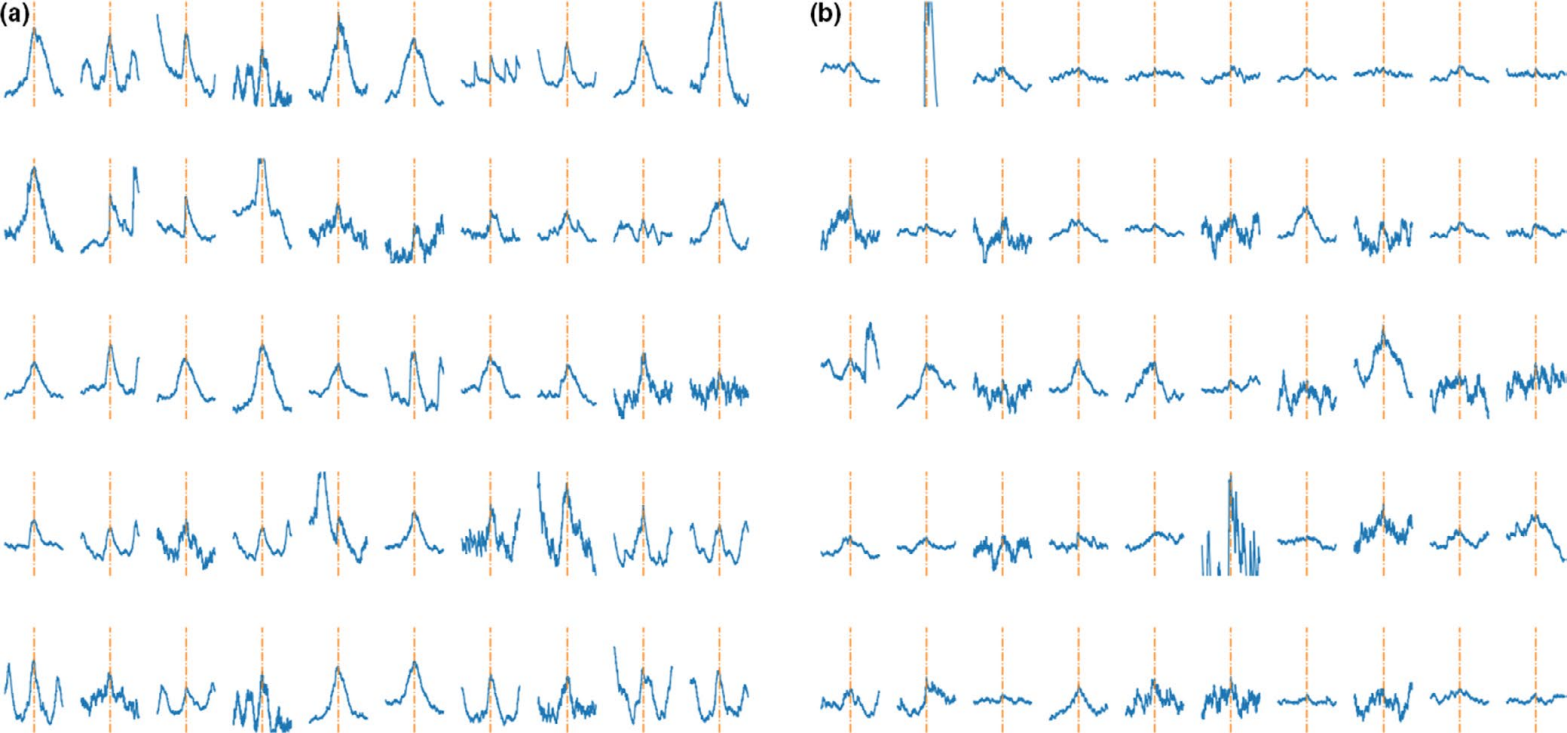
Examples from the labeled dataset for the second neural Network DTCT 2. A shows signal peaks that were rated to likely represent valid MSNA signals. b, shows examples of signal peaks rated not to represent likely valid MSNA signals. The signal is orientated in a way that its highest point is the center of the image and additionally marked with an orange, interrupted line

**FIGURE 6:**
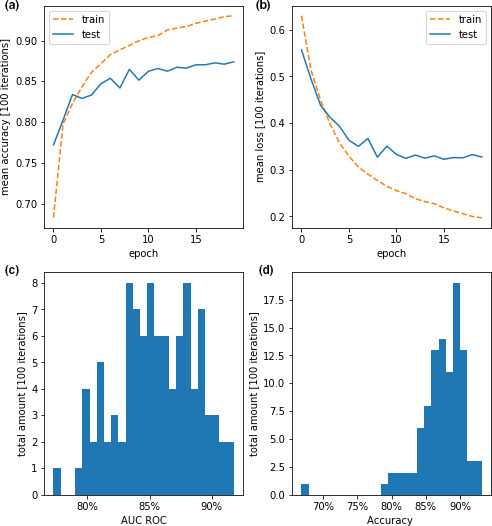
Performance parameters for the training for DTCT 2. Panel (a) and (b) show the average accuracy and loss of 100 individual training iterations of the algorithm for training and testing dataset. Panels (c) and (d) show histograms of the performance parameters AUC ROC and accuracy for each individual time the algorithm was trained

Examples of the labeled dataset that was used for training of the algorithm are shown in Figure 5. Over 100 training runs of the algorithm resulted in a mean accuracy of 87.4% and a mean AUC of the ROC of 86%. Summarizing graphs of the trained algorithms are displayed in Figure 6–with the average learning curves and histograms for the distribution of the testing parameters accuracy and AUC of the ROC.

#### 3.1.3 Third neural network DTCT 3

**FIGURE 7:**
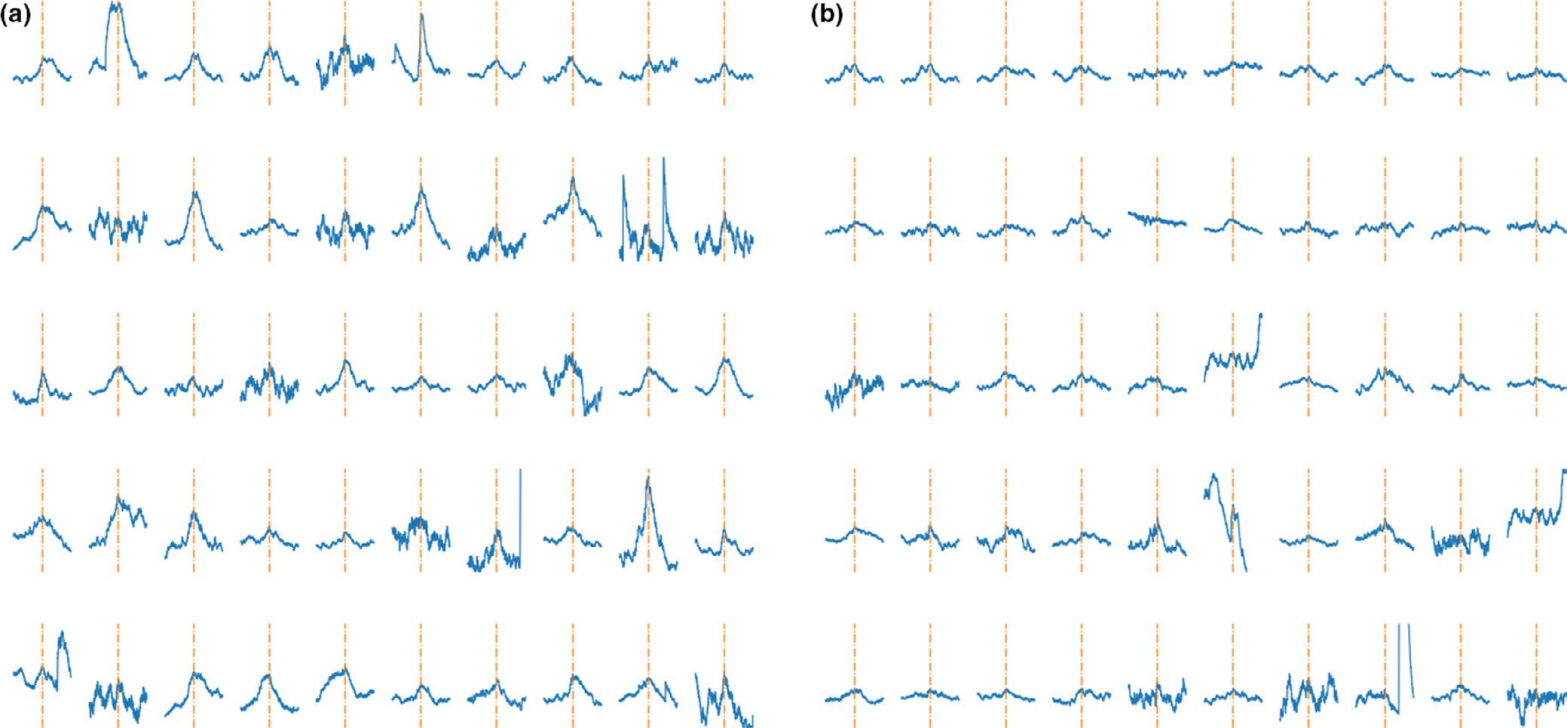
Examples from the labeled dataset for the third neural Network DTCT 3. Panel (a) shows signal peaks that were rated to likely represent valid MSNA signals. (b) shows examples of signal peaks rated not to represent likely valid MSNA signals. The signal is orientated in a way that its highest point is the center of the image and additionally marked with an orange, interrupted line

**FIGURE 8:**
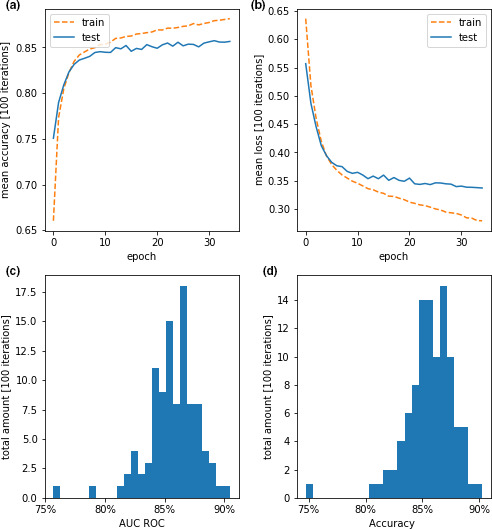
Performance parameters for the training for DTCT 3. Panels (a) and (b) show the average accuracy and loss of 100 individual training iterations of the algorithm for training and testing dataset. Panels (c) and (d) show histograms of the performance parameters AUC ROC and accuracy for each individual time the algorithm was trained

Examples of the labeled dataset used to train the third neural network are visualized in Figure 7 separated for positively rated signals and negatively rated signals–each representing likely valid and non‐valid signal peaks, respectively. The 100 iterations of training for the DTCT 3 resulted in an average accuracy of 85.8% and an average AUC of the ROC of 86%. Performance parameters for DTCT 3 are visualized in Figure 8.

### 3.2 Signal timing–association of MSNA‐Signals, Blood pressure and ECG

The median difference between the local BP minima and the corresponding ECG maxima (R‐wave) was −179 ms in the final selected distribution is shown in Figure 7a. Before the selection process by the means of an assigned z‐score and clipping the data at −250 ms, the distribution had a mean of −182 ms–an excerpt of this distribution is shown in Figure 7b (distribution after z‐score clipping, the original distribution had extreme values up to −30,000 ms).

### 3.3 Automated Markers for signal quality quantification

Of the 60 s raw data excerpts (plus and minus 30 s) around each MSNA signal candidate a simple one‐dimensional discrete Fourier transformation was calculated. The median value of these values for each candidate MSNA signal was plotted during the time course of MSNA recordings. For a visualization of the transformation process see Figure 2, for a visualization of the median values of the transformed data around each candidate signal see Figure 10.

**FIGURE 9:**
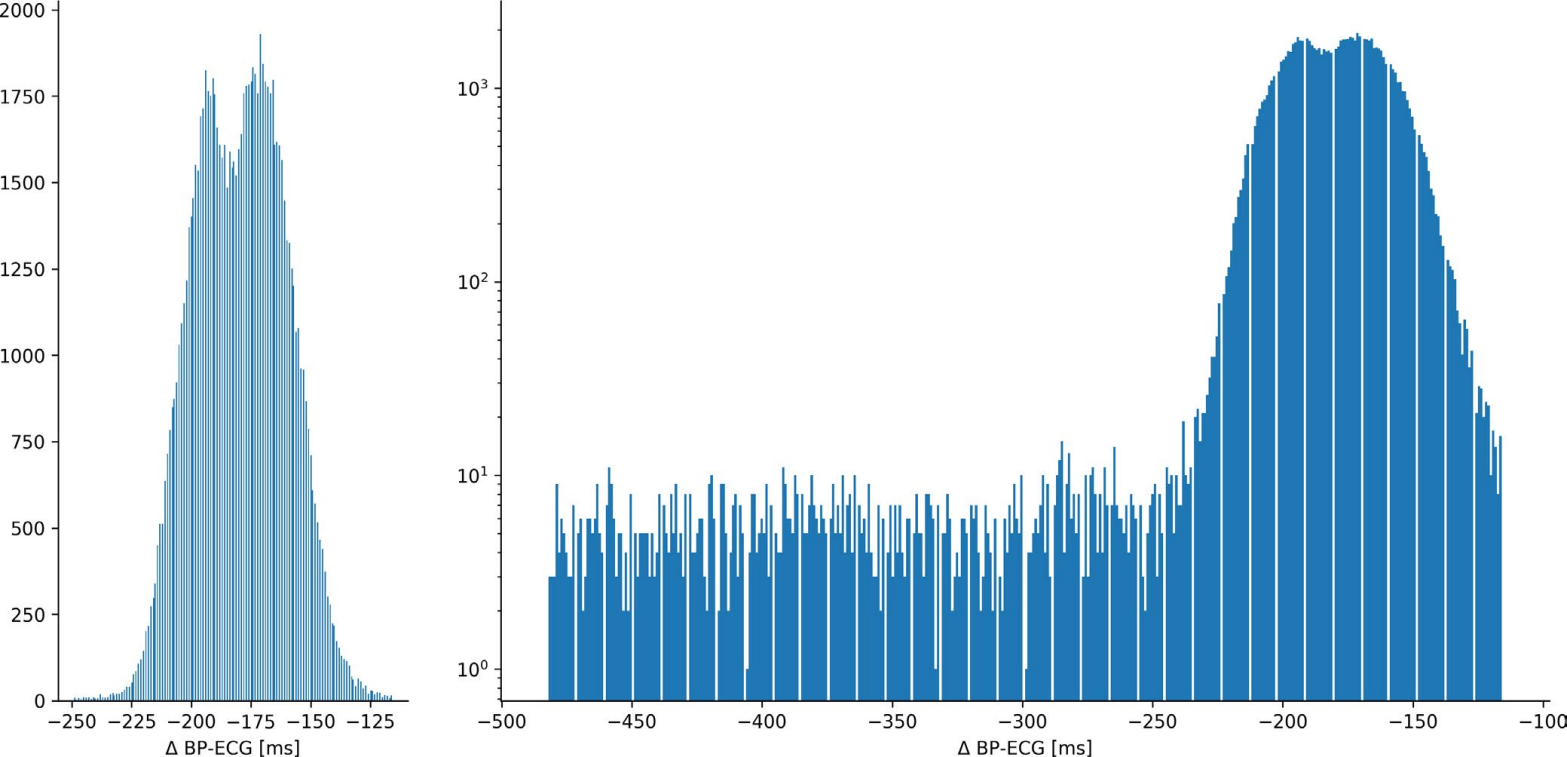
Distribution of time difference between ECG‐peaks (R‐wave) and lowest blood pressure signal. Panel (a) shows the final, focused distributions, and panel (b) (log‐scale) shows the distribution with long left, negative tail after z‐score based data selection and before clipping of extreme values below −250 ms

**FIGURE 10:**
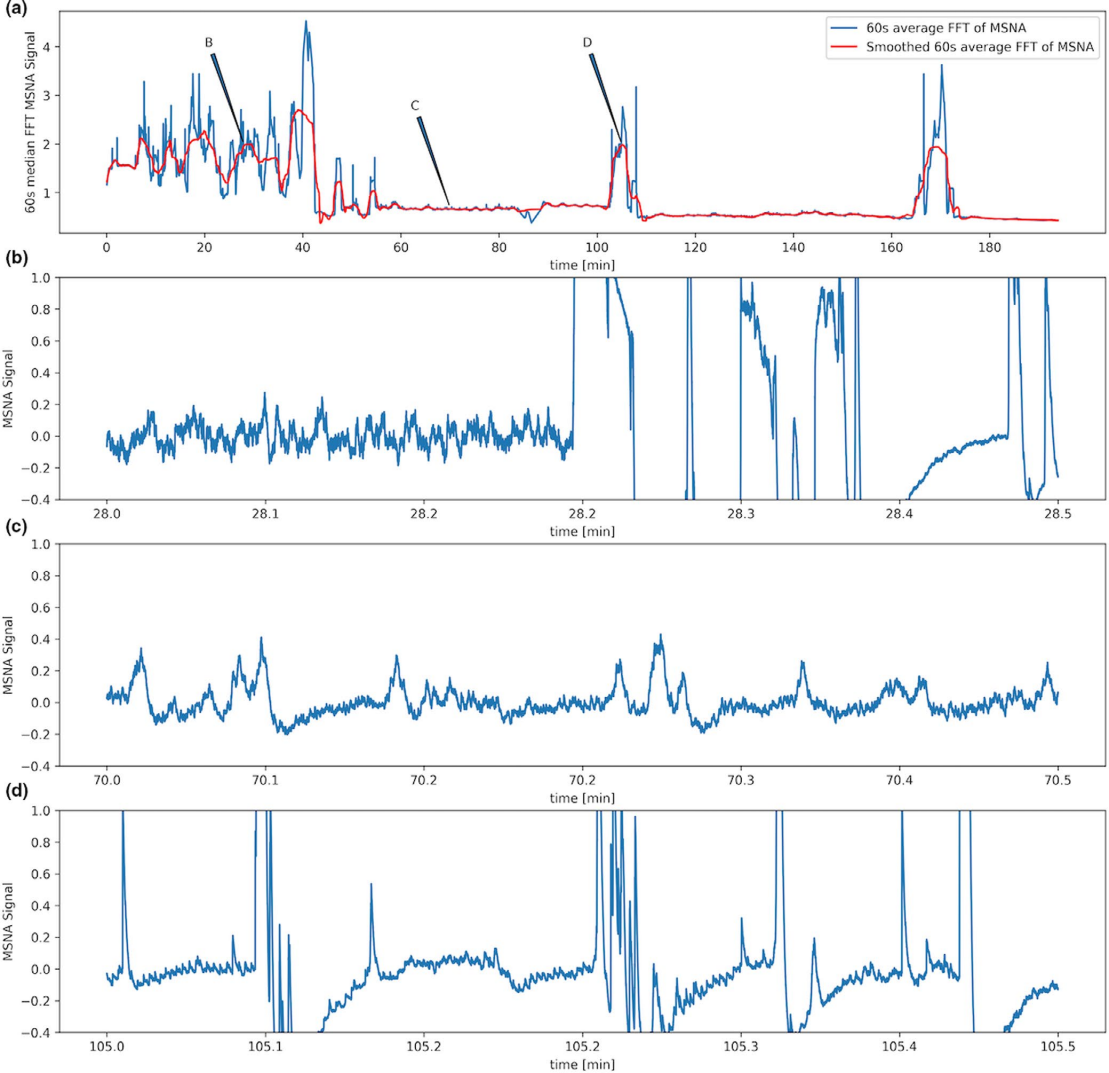
(a) shows the quality index derived from the Fourier transformed of a 3‐h long microneurography recording. The quality index is calculated for each detected signal peak during this time for a 60‐s window around the peak. The median of the Fourier transformation for these excerpts is associated with the individual peaks and plotted over time (a). Low, relatively constant intervals of this variable represent stable and valid MSNA recordings, higher and varying levels periods of positioning the needle or losing the signal for example after the participant moved. The excerpts at points (b, c and d) have been chosen randomly based on this quality index. Point (b) is located at a time point with high variance and generally high levels of the quality marker–indicating bad signal quality. The sub‐panel (b) shows the original microneruography signal corresponding to this time point–showing a noisy, non‐valid signal. At time point (c) the quality index suggest a stable, valid signal–as seen in the corresponding original data window in sub‐panel (c). At time point (d) the quality index has suddenly sored upwards again, indicating a loss of valid signal, confirmed by the corresponding original data in sub‐panel (d)

**FIGURE 11:**
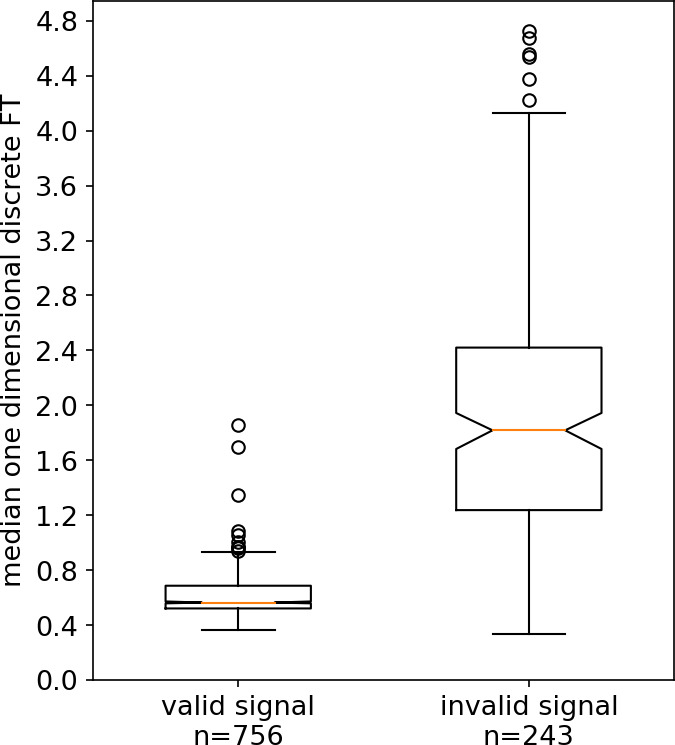
Boxplot showing the distribution of median values of the one dimensional Discrete Fourier transformation (DFT) for 990 excerpts of MSNA recordings. These recordings are rated into valid and invalid recordings–analysis was carried out according to this grouping of the data. The middle bar of the box represents the median, borders of the boxes represent the interquartile ranges, notches the bootstrapped 95% confidence intervals. Whiskers and circles are showing outliers of the data

On careful inspection of the time course of this parameter during a microneurography recording in various subjects, distinct and sudden changes of this parameter indicated changes in signal quality, whereas stable time periods with values below indicated a valid acquisition of the MSNA signal. Examples of this are shown in Figure 10. A dataset of 1000 signal excerpts of 10 different MSNA recordings of different subjects, which were rated in terms of validity of the signal, were assessed making use of this parameter. Results are shown in the boxplots of Figure 11. The median value of the group of data that was rated to represent valid signal recordings was 0.57 (*n* = 757 excerpts). In the group that was rated not to represent a valid MSNA recording the median was 1.82 (*n* = 233 excerpts), a Mann‐Whitney *U*‐test estimated for the differences between the groups a p‐value <0.001 (U = 13723).

### 3.4 Implementation

**FIGURE 12:**
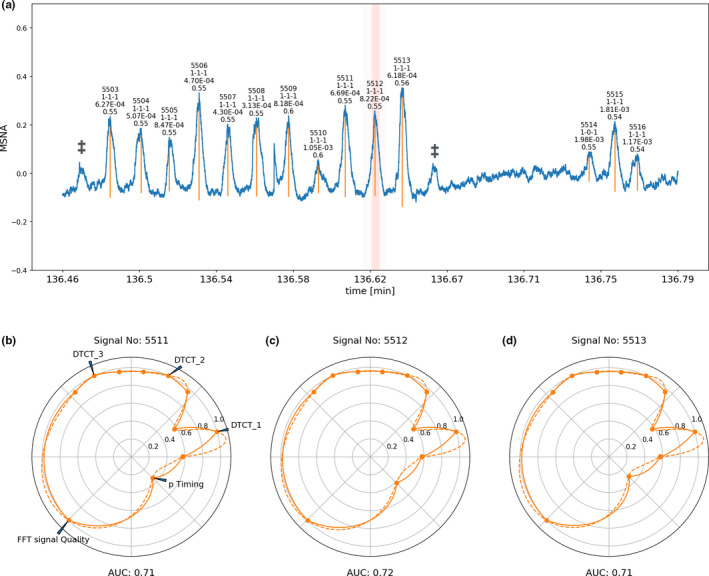
Signal window of microneuroggraphy recording of excellent quality. Signal peaks selected by the software are marked with vertical orange lines. The most important variables assigned to each detected signal peak are printed on top of each individual peak (first line consecutive signal number, second line binarized output of the three neural networks with 1 indicating valid signal and 0 non‐valid signal, third line absolute probability density of the individual peaks based on the timing model, last line median of the Fourier transformed 60‐s window around the signal). (b–d) are circular representations of the most important determined variables for each individual peak, subpanel (c) corresponds to the central peak marked in red in subpanel (a), the circles right and left to the corresponding next signal peak right and left of the marked central peak. The circular graphs visualizes the binary decision of the individual DTCT neural network algorithms (which are marked as DTCT_1, DTCT_2, and DTCT_3) surrounded each left and right by the continuous likelihood for a valid signal given out by the respective algorithm. The overall quality index (FFT signal Quality) rescaled from 0 to 1 (1 indicating an excellent signal) is plotted in the left bottom corner, the rescaled probability calculated based on the timing of the signal (p‐timing, 1 indicating high likelihood) is plotted in the right bottom corner. Calculation of the area under the curve from this plot and rescaling of the AUC to 0 to 1 yields an overall integrated likelihood for the signal to be and valid MSNA signal. Some signals have been marked with ‡ to indicate that the first identification step of candidate signal burst with conventional peak detection algorithms at times left out signals that may be regarded by some to be valid candidate signals. These signals however, are in turn not considered by our more advanced signal processing algorithms

**FIGURE 13:**
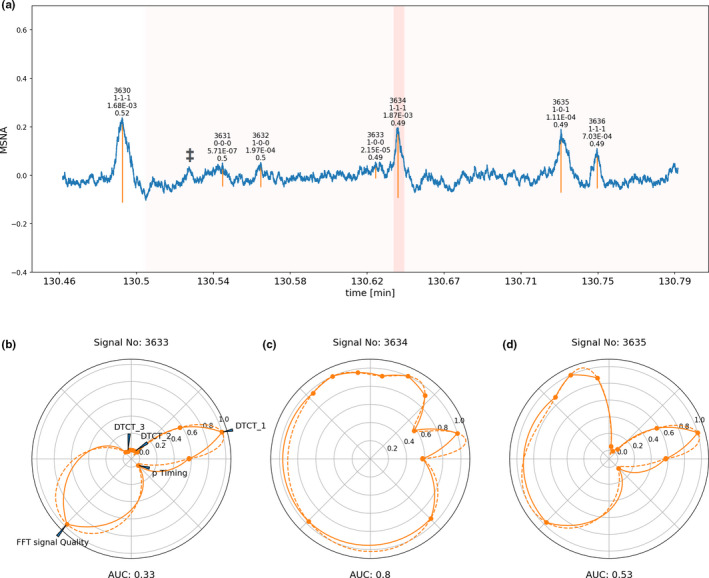
Signal window of microneuroggraphy recording of average quality. Signal peaks selected by the software are marked with vertical orange lines. The most important variables assigned to each detected signal peak are printed on top of each individual peak (first line consecutive signal number, second line binarized output of the three neural networks with 1 indicating valid signal and 0 non‐valid signal, third line absolute probability density of the individual peaks based on the timing model, last line median of the Fourier transformed 60‐s window around the signal). (b–d) are circular representations of the most important determined variables for each individual peak, subpanel (c) corresponds to the central peak marked in red in subpanel (a), the circles right and left to the corresponding next signal peak right and left of the marked central peak. The circular graphs visualizes the binary decision of the individual DTCT neural network algorithms (which are marked as DTCT_1, DTCT_2 and DTCT_3) surrounded each left and right by the continuous likelihood for a valid signal given out by the respective algorithm. The overall quality index (FFT signal quality) rescaled from 0 to 1 (1 indicating an excellent signal) is plotted in the left bottom corner, the rescaled probability calculated based on the timing of the signal (p‐timing, 1 indicating high likelihood) is plotted in the right bottom corner. Calculation of the area under the curve from this plot and rescaling of the AUC to 0 to 1 yields an overall integrated likelihood for the signal to be and valid MSNA signal. Some signals have been marked with ‡ to indicate that the first identification step of candidate signal burst with conventional peak detection algorithms at times left out signals that may be regarded by some to be valid candidate signals. These signals however are in turn not considered by our more advanced signal processing algorithms

**FIGURE 14:**
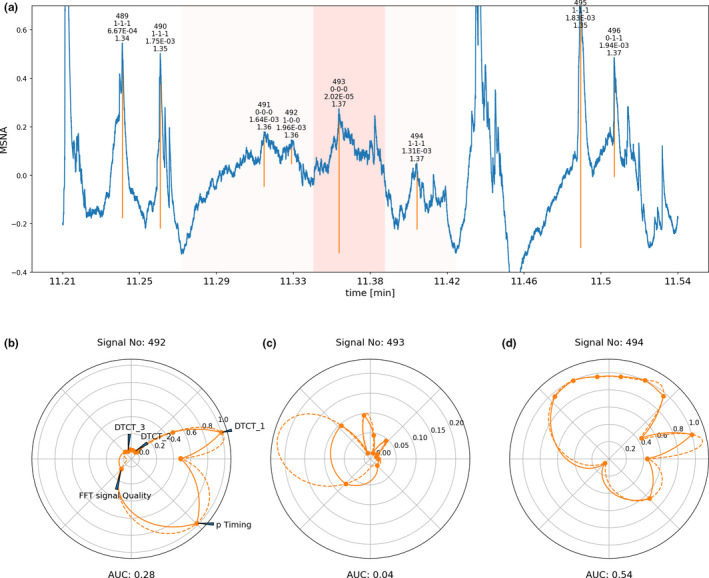
Signal window of microneuroggraphy recording of poor quality. Signal peaks selected by the software are marked with vertical orange lines. The most important variables assigned to each detected signal peak are printed on top of each individual peak (first line consecutive signal number, second line binarized output of the three neural networks with 1 indicating valid signal and 0 non‐valid signal, third line absolute probability density of the individual peaks based on the timing model, last line median of the Fourier transformed 60‐s window around the signal). (b–d) are circular representations of the most important determined variables for each individual peak, subpanel (c) corresponds to the central peak marked in red in subpanel (a), the circles right and left to the corresponding next signal peak right and left of the marked central peak. The circular graphs visualizes the binary decision of the individual DTCT neural network algorithms (which are marked as DTCT_1, DTCT_2, and DTCT_3) surrounded each left and right by the continuous likelihood for a valid signal given out by the respective algorithm. The overall quality index (FFT signal quality) rescaled from 0 to 1 (1 indicating an excellent signal) is plotted in the left bottom corner, the rescaled probability calculated based on the timing of the signal (p‐timing, 1 indicating high likelihood) is plotted in the right bottom corner. Calculation of the area under the curve from this plot and rescaling of the AUC to 0 to 1 yields an overall integrated likelihood for the signal to be and valid MSNA signal

Each step of assessment described above was carried out for each signal‐peak detected in the MSNA recording. The implemented data were visualized in panels for each signal‐peak including an overall likelihood estimate. Examples of these visual representations can be seen in Figures 12, 13, 14 in MSNA signal of different quality.

## 4 DISCUSSION

MSNA signals are difficult to obtain, signal quality varies widely and recordings tend to be noisy and contain many artifacts. Analysis is therefore often performed manually or with simplistic peak‐detection algorithms that are customized individually and applied to selected, high quality windows of the recordings. Considerable efforts have been made to standardize approaches (White et al., 2015). However, manual analysis often remains subjective and to a certain degree difficult to standardize, especially with lower quality signals. Peak detection software approaches can often be helpful for good quality excerpts, which need to be selected in a subjective manual approach.

The longer the presented microneurography excerpt, the more will the rating take into account the overall signal quality. The shorter the presented window, the more will the focus shift on only the form of the individual localized peaks. Given that we wanted to create an additional, independent parameter for the overall signal quality, we chose for this implementation short excerpts of 1 and 4 s.

Software capable of detecting periods of valid recordings automatically on longer recording excerpts and deciding which signal peaks in the shorter recording excerpts are likely correspond to MSNA may be a possibility to make the analysis of microneurography more efficient, objective, and reproducible and therefore more standardized.

We have described novel software tools based on artificial intelligence, machine learning, and probabilistic models that reproduce manual decisions about MSNA signals. We believe that the accuracy is highly dependent on coherent labeling and inaccuracy of predictions often driven by in‐between cases of signals that might be interpreted as valid or invalid given certain circumstance, for example signal quality and timing. Since the overall model takes these factors into account the overall analysis of recordings will likely perform even better.

Another point to consider is the missing ground truth in the labeling of these signal‐peaks, which remains manual and subjective and therefore a gold‐standard method at best. Future research will need to look into the possibility of overcoming this fallacy of reproducing manual decisions and reaching a more standardize approach in classifying these signals–potentially based on unsupervized automated learning approaches. However, the approach to evaluate signals with a software–even based on subjective manual decisions–makes the decisions from this point on coherent and reproducible, which is the clear advantage of using automated methods–besides increasing efficiency. The application can be individualized to the preferences of the user by building and adding custom trained neural networks based on data labeled by the user. This would reproduce the decisions and preferences of individual users. This does, however, not mitigate the requirement for further research to employ rigorous validation techniques in this context to further define the relationship between the described approaches and manual analyses of MSNA signals.

The presented evidence of the regular connection between ECG and continuous blood pressure opens up the possibility to use either of these to evaluate the timing of the signals by just adjusting the assumed time delay by 180 ms. Blood pressure is the more directly related marker physiologically, while ECG is often more stable and less prone to show artifacts. Taking into account the time it takes for afferent sensory signals to reach autonomic centers of the brain, be processed and travel via slow C‐fibers to the site where the MSNA can be obtained via the recording electrode, there is a constant temporal relationship between continuous blood pressure, ECG, and likelihood of an MSNA burst occurrence during the cardiac cycle (Delius et al., 1972; White et al., 2015). This relationship can help to identify valid MSNA bursts and differentiate them for example from skin nerve bursts–which have a more variable appearance and lack this cardiac rhythmicity (Gunnar, 2007; Hagbarth et al., 1972).

Depending on which data were recorded and in which quality will enable examiners to decide which one to use for the analysis, and a flexible platform allowing multiple approaches to determine if an MSNA burst is valid or not would enable customization of approaches and preferred algorithms. The disadvantage of personalizing these systems would obviously be that it diminishes one of the great advantages of the technology, which is to standardize MSNA analysis between different laboratories. If interpreters would stick to a default version of the software analysis of MSNA data would henceforth only be determined by the raw data and the software version of the analyzing algorithm.

After application of the described methods, the algorithm produces a dataset with a large number of features and validity likelihoods for each signal and integrates them in one value. Signals, from this point on, can be selected with a default likelihood of >0.5 or with stricter or less strict criterion with the possibility of adjusting the weighting of the factors determining the overall likelihood (by controlling how much width under the curve of the final circular summarizing graphs they occupy). Furthermore, the algorithm can be adjusted so that only valid signal periods are taken into account referring to the quality parameters (which is also by default part of the overall likelihood). Standard parameters like bursts per minute or per heart beat as well as total markers like product of burst frequency and mean burst height can be easily extracted from valid recording excerpts (White et al., 2015). New markers will be ready to be created and computed as the data is fully digitalized and implemented in environments that make further exploration feasible.

Our approach has several limitations: Conventional peak detection software may not be set up to be sufficiently inclusive in its choice of candidate signal bursts, such that further evaluated relevant signal burst may be missed entirely. We have marked some examples that could be considered to represent valid MSNA bursts by some interpreters (Figures 12 and 13) to showcase this possibility. This potential limitation can be ameliorated by choosing a setting for the conventional peak detection algorithms that is sufficiently broad. This, however, may have to be balanced against the potential he disadvantage of decreasing the pre‐test probability of choosing valid signals, which may have an impact on overall performance of the methods following initial signal candidate selection in the pipeline.

Our description of tools to facilitate automated approaches for MSNA analysis, while portraying quantifications of the performance of individual components, does not contain a fully validated approach. Implementation of these tools into an analysis algorithm will require further rigorous validation of the method and comparison with current gold‐standard manual analysis. Further limitations of this work include the lack of the full quantification of the MSNA signals manually and automatically and comparison thereof, which will be required for future validation of the approach, as well as for demonstration of the suitability of the data used for algorithm generation to represent a wide range of baseline sympathetic activity patterns.

For the current research, microneurography recordings from people with a diverse demographic were chosen to facilitate broader representation. However, we only included male participants at this stage to limit any potential sex bias, thereby limiting its applicability to female sex. This however, could be easily overcome by the addition of further prediction algorithms that are trained on mixed sex or female only datasets.

Another target of future research may be the exploration of more sophisticated models to capture the temporal association between valid MSNA signals and the cardiac cycle. While all microneurography recordings utilized in the presented work were derived from resting recordings, sympathetic stressors and task‐related MSNA may lead to more variability in this temporal association and require more advanced modelling approaches.

In summary, we have introduced a number of novel tools with promising potential to automate the analysis of microneurography recordings.

## CONFLICTS OF INTEREST

JMN and the other authors declare that they have no conflict of interest. LMLG has received a scholarship from the National Council on Science and Technology, Mexico (CONACYT). RC is supported by the Australian National Heart Foundation post doc fellowship. MPS is supported by an NHMRC Research Fellowship and has received consulting fees, and/or travel and research support from Medtronic, Abbott, Novartis, Servier, Pfizer, and Boehringer‐Ingelheim.

## AUTHOR CONTRIBUTIONS

Janis M Nolde: Conceptualization, Data Collection, Data curation, Algorithm Development, Computation, Formal analysis, Visualization, Writing–original draft, Writing–review & editing; Leslie Marisol Lugo‐Gavidia: Data Collection, Data curation, Writing–review & editing; Revathy Carnagarin: Data Collection, Writing–review & editing; Omar Azzam: Data Collection, Writing–review & editing; Márcio Galindo Kiuchi: Data Collection, Writing–review & editing; Ajmal Mian: Supervision, Conceptualization, Algorithm Development, Computation, Formal analysis, Visualization, Writing–review & editing; Markus P Schlaich: Supervision, Conceptualization, Data Collection, Formal analysis, Visualization, Writing–review & editing.

## Data Availability

The data underlying this article cannot be shared publicly due to the privacy of individuals that participated in the study.
